# Glucose Induces IL-1α-Dependent Inflammation and Extracellular Matrix Proteins Expression and Deposition in Renal Tubular Epithelial Cells in Diabetic Kidney Disease

**DOI:** 10.3389/fimmu.2020.01270

**Published:** 2020-07-07

**Authors:** Talal Salti, Khaled Khazim, Rami Haddad, Salvatore Campisi-Pinto, Gil Bar-Sela, Idan Cohen

**Affiliations:** ^1^Galilee Medical Center, Research Institute, Nahariya, Israel; ^2^Azrieli Faculty of Medicine, Bar-Ilan University, Safed, Israel; ^3^Department of Nephrology and Hypertension, Galilee Medical Center, Nahariya, Israel; ^4^Bruce Rappaport Faculty of Medicine, Technion-Israel Institute of Technology, Haifa, Israel; ^5^Cancer Center, Emek Medical Center, Afula, Israel

**Keywords:** interleukin 1, diabetic nephropathy (DN), inflammation, kidney, alarmins, stressorin, extracellular matrix

## Abstract

Diabetes mellitus is linked with metabolic stress that induces cellular damage and can provoke renal inflammation and fibrotic responses that eventually lead to chronic kidney disease. Because the inflammasome, interleukin 1 (IL-1), IL-1α/IL-β, and IL-1R are central elements of kidney inflammation and pharmacological IL-1R antagonist (IL-1Ra) was shown to prevent or even reverse diabetic nephropathy (DN) in animal models, we explored the intrinsic expression of IL-1 molecules in kidney tissue of DN patients as regulators of renal inflammation. We used biopsies taken from DN patients and controls and show a high level of IL-1α expression in renal tubular epithelial cells, whereas both IL-1 agonistic molecules (i.e., IL-1α and IL-1β) were devoid of the glomeruli. Human proximal tubular kidney HK-2 cells exposed to high glucose (HG) gradually increase the expression of IL-1α but not IL-1β and induce the expression and deposition of extracellular matrix (ECM) proteins. We further demonstrate that *in vitro* ectopic addition of recombinant IL-1α in low glucose concentration leads to a similar effect as in HG, while supplementing excess amounts of IL-1Ra in HG significantly attenuates the ECM protein overexpression and deposition. Accordingly, inhibition of IL-1α cleaving protease calpain, but not caspapse-1, also strongly reduces ECM protein production by HK-2 cells. Collectively, we demonstrate that IL-1α and not IL-1β, released from renal tubular cells is the key inflammatory molecule responsible for the renal inflammation in DN. Our result suggests that the clinical use of IL-1Ra in DN should be promoted over the individual neutralization of IL-1α or IL-1β in order to achieve better blocking of IL-1R signaling.

## Introduction

Diabetic nephropathy (DN) or diabetic kidney disease (DKD) is one of the most frequent complications of both types of diabetes mellitus (1), affecting ~40% of diabetic patients (2). Diabetic kidney disease is the leading cause of end-stage renal disease (ESRD) (3), accounting for almost 50% of ESRD cases in developed countries (1). Diabetic kidney disease is also highly associated with an increased risk of cardiovascular mortality (2, 3), imposing a massive social and economic burden on affected individuals and the health care systems (4).

Diabetic kidney disease is clinically defined as progressively increasing proteinuria accompanied by increasing blood pressure and impairment of glomerular filtration. Histologically, it is characterized by diffuse or nodular glomerulosclerosis and afferent and efferent hyaline arteriolosclerosis (5). Although glomerulosclerosis is a cardinal future of DKD, the extension of the tubulointerstitial injury determines the rate of attrition of renal function (6). Proximal tubular dysfunction, in addition to increased glomerular leakage, is potentially responsible for microalbuminuria in early-stage DKD (7). “Diabetic tubulopathy” is an emerging entity that suggests a role of tubular injury in the pathogenesis of DKD. Numerous potential damaging agents may cause tubular cells injury by triggering a proinflammatory and profibrotic response (8).

Currently, several evidences suggest that inflammation-related molecules and pathways play a central role in DKD (9). Of late, proinflammatory cytokines such as interleukin 1 (IL-1), IL-6, IL-18, or tumor necrosis factor α; damage-associated molecular patterns (DAMPs) or alarmins; and the inflammasome complex (10, 11) were found to be involved (9, 12).

Interleukin 1 is a master cytokine of local and systemic inflammation. Locally, IL-1 triggers vasodilation attracts granulocytes to the site of tissue damage and stress. Interleukin 1 family consists of two proinflammatory cytokines, IL-1α and IL-1β, and a naturally occurring anti-inflammatory agent, the IL-1Ra (13, 14). Interleukin 1α and IL-1β are produced by both blood-borne immune cells as well as by intrinsic renal cells as it was observed in human proliferative and non-proliferative forms of glomerulonephritis where there was increased production of IL-1 in the podocytes (15). Likewise, it was demonstrated that there is an intrinsic overexpression of IL-1 in animal models suffering from DN, and the application of a pharmacological IL-1Ra proved to prevent the progression and even reverse DN in an animal model (16). Yet, the interaction between these factors, the particular IL-1 cytokine, and its primary cell source, which triggers the renal inflammation in DKD, remains unknown. Here we explored the exact pathophysiology behind DKD and showed high levels of IL-1α rather than IL-1β expression in human tubular epithelial kidney cells are most likely responsible for the IL-1–dependent inflammation in DKD.

## Methods

### Study Cohort

This is a retrospective study that was conducted at the Galilee Medical Center, Naharyia, Israel, after the approval of the Helsinki Committee (protocol no. 0067-17 NHR). The research includes two groups of patients. Group 1 consists of six type 2 diabetic patients who had undergone kidney biopsy due to clinical indications, and the histological analysis showed DKD. The second group or control group includes six non-diabetic patients who had undergone elective nephrectomy due to renal carcinoma and did not have any form of inflammation in their kidney tissue when they had their pathological examination. Exclusion criteria were as follows: histological signs of inflammation or DKD for the control group; histological signs of glomerulonephritis, interstitial nephritis, or any other pathological changes in the kidney tissue of both groups. Data collection did not include direct contact with the patients. Diabetic kidney disease patients' clinical and laboratory data [age, sex, glomerular filtration rate (GFR), urine protein, serum albumin, total serum protein, blood urea nitrogen, and creatinine levels] were collected corresponding to the closest date from the renal biopsy and are summarized in Table 1.

**Table 1:** Patients parameters.

**Gender**	**Age**	**GFR [ml/min/1.73 me2]**	**Urine protein [g/24 h]**	**Mark**
Female	47	44	8	DN1
Female	48	21	5.1	DN2
Male	66	45	13	DN3
Male	41	27	4.47	DN4
Male	71	40	19	DN5
Female	59	13	10	DN6
**Gender**	**Age**	**eGFR**		**Mark**
Female	79	85		C1
Female	64	95		C2
Female	30	120		C3
Male	46	76		C4
Female	61	62		C5
Female	67	95		C6

### Immunohistochemical and Immunofluorescent Staining

Immunohistochemical procedure was performed on 4-μm consecutive sections of human kidney paraffin-embedded sections. Deparaffinization was performed by washing the slides with specific reagents in the following order: xylene 2× for 5 min, 100% ethanol 2× for 5 min, and slides were then transferred once through 95, 70, and 50% alcohols, respectively, for 5 min each time and finally rinsed with distilled water 2× min. For blocking the endogenous peroxidase activity, we incubated the sections in 3% H_2_O_2_ solution at room temperature for 10 min. The slides were then rinsed with 50 μM Tris-buffered saline (TBS) pH 7.6 for 5 min before antigen retrieval with citrate buffer pH 6, at 95°C for 30 min. Samples were brought to room temperature and rinsed twice with TBS for 5 min. The slides were covered with diluted normal serum for 10 min. Primary antibodies [e.g., monoclonal antihuman IL-1α (MAB200-100; R&D Systems, Minneapolis, USA) and monoclonal antihuman IL-1β antibody (MAB601-100; R&D Systems) in ~1:50 final dilutions (vol/vol) were applied and incubated at 37°C for 30 min. Samples were washed in TBS twice for 5 min with gentle rocking, followed by application of horseradish peroxidase goat antimouse immunoglobulin G (IgG) secondary antibody (ab6789; Abcam) for 8 min at 36°C. We rewashed the slides 2× with TBS 5 min with gentle rocking and added DAB-H_2_O_2_ for 8 min at room temperature followed by washing with dH_2_O. The sections were counterstained with hematoxylin and then rinsed with running tap water, dehydrated, and eventually mounted with a mounting solution (Tissue-Tek Glas mounting media; Skura, Alphen aan den Rijn, Netherlands). Slides were scanned and documented using Nikon eclipse I90 with a CCD camera microscope, Tokyo, Japan. The immunostaining intensities were measured using Nikon NIS elements Ver4 software and were evaluated in a double-blind manner.

The immunofluorescent (IF) protocol follows the previously published IF protocol for IL-1α (17, 18). Briefly, mouse monoclonal antifibronectin (sc8422) and mouse monoclonal anti–α-SMA (sc53142) were used in 1:100 dilution following by staining with a donkey antimouse IgG DyLight 488 (Bethyl A90-337D2) and were visualized with a wide-field fluorescence microscope (Nikon Eclipse).

### Cell Culture Inhibitors and Reagents

The HK-2 renal proximal tubular epithelial cell line was kindly gifted by Prof. Karl Skorecki (Technion, Haifa, Israel) and was obtained from the American Type Culture Collection (ATCC, Manassas, VA, USA). The cells were cultured in Dulbecco modified eagle medium (DMEM) supplemented with 2 mM l-glutamine, 10% fetal bovine serum (FBS) (Sigma–Aldrich, St. Louis Germany, USA), penicillin/streptomycin, and 1% insulin–transferrin–selenium (Gibco, Waltham, United States) and were cultured in 37°C with 5% CO_2_. For *in vitro* glucose assays, 1 × 10^6^ HK-2 cells (per well) were made quiescent by preincubation in serum-free DMEM/F12 medium with 5 mM glucose for 24 h; 5.5 mmol/L was regarded as normal glucose concentration, whereas 30 or 45 mM glucose was chosen to investigate the mechanism underlying HG-induced cell injury in DMEM/F12 medium containing 1% FBS. No substantial decrease in cell viability was detected during these periods with this protocol of studying HG effect of HK-2 cells, as previously described in Zhou et al. (19). Recombinant human IL-1Ra (IL-1RN) (carrier-free) (BLG-553902) and recombinant human IL-1α (hIL-1α) (carrier-free) were both purchased from Bioligand, San Diego, United States (BLG-570002) and were used as indicated in the range of 0.1 up to 10 ng/mL. l-Glucose and d-glucose were both purchased from Santa Cruz Biotechnology, Santa Cruz, California, United States, and the nuclear factor κB (NF-κB) inhibitor BAY 11-7082 was purchased from Cayman Chemical, Ann Arbor, MI, United States (cat. no. 10010266). The caspase-1 inhibitor Ac-YVAD-cmk (cat. no. 178603-78-6 in final concentration of 50 μM) and the calpain inhibitor calpeptin (cat. no. 117591-20-5 in final concentration of 20 mM) were both from Cayman Chemical.

### Quantitative Real Time–Polymerase Chain Reaction

Total RNA was extracted from three consecutive whole sections (8 μm) of formalin-fixed kidney biopsies containing both renal tubules and glomeruli with no laser dissections using the ReliaPrep FFPE Total RNA Miniprep system (Promega, Madison, Wisconsin, United States). Total RNA 100 ng was converted to cDNA using the reverse transcription (RT) system High-Capacity cDNA Reverse Transcription Kit (AB; Thermo, Waltham, United States) according to the manufacturer's instructions. Real-time quantitative RT–polymerase chain reaction (qPCR) was performed using the StepOnePlus real-time PCR System (Applied Biosystems, Waltham, United States) with Fast SYBR® Green Master Mix (Thermo Fisher Scientific, Waltham, United States).

For *in vitro* metabolic and chemical inhibition assays using HK-2 cells, total RNA was extracted using the RNeasy Mini Kit (Qiagen, Hilden, Germany) (~1 × 10^6^ cells per well). Total RNA 1,000 ng was converted to cDNA using the RT system High-Capacity cDNA Reverse Transcription Kit (AB; Thermo) according to the manufacturer's instructions. Real-time qRT-PCR was performed using the StepOnePlus real-time PCR System (Applied Biosystems) with Fast SYBR® Green Master Mix (Thermo Fisher Scientific). Primers used in this study are listed in (Supplementary Table 1).

### Western Blot Antibodies and Recombinant Proteins

Total proteins from the renal proximal tubular epithelial HK-2 cells were extracted with 400 μL of RIPA buffer (Sigma) from 1 × 10^6^ cells per well. The suspensions were incubated 10 min on ice, centrifuged for 10 min at 12,000 g to remove insoluble debris, and surfactants were mixed with 1× Laemmli buffer and built for 10 min. Equal protein loads were separated over 10 or 15% sodium dodecyl sulfate–polyacrylamide gel electrophoresis. Protein levels in the conditioned medium or the cell lysates were determined by immunoblotting with the following antibodies: mouse monoclonal antifibronectin (sc8422), mouse monoclonal anti–α-SMA (sc53142), and mouse monoclonal antiactin (8H10D10) (Cell Signaling #3700, Danvers, Massachusetts, United States), which was used as a loading control.

### Statistical Analysis

We used a one-way analysis of variance to test how likely it was that any observed difference between the groups arose by chance. For each test, the null hypothesis (H_0_) was set to zero. We tested H_0_ using sample data. Conventional *p*-value thresholds (^*^p < 0.05, ^**^p < 0.01, and ^***^p < 0.001) were used to rejected H_0_ in favor of the alternative hypothesis and to report the significance level of the difference between groups. Results are presented as the mean of a minimum of three independent experiments ± SD. Statistical significance was determined by *t*-test comparing two different samples.

## Results

**Figure 1:**
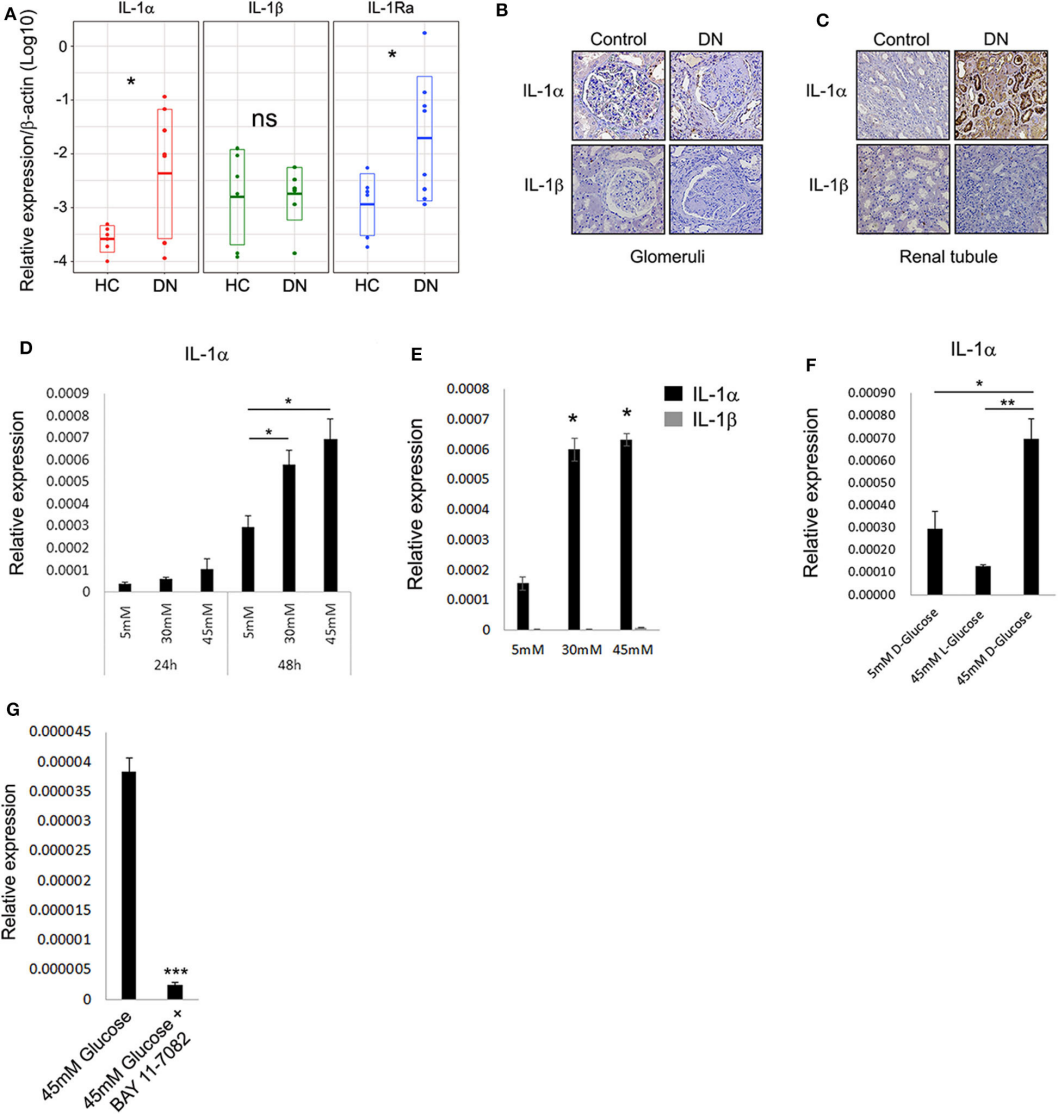
Interleukin 1α is highly expressed in renal tubular epithelial cells of patients with diabetic kidney disease. **(A)** Gene expression data of IL-1 genes transcripts: IL-1α, IL-1β, and IL-1Ra. Total RNA extracted from paraffin-embedded whole sections of kidney biopsies of DN and HC donors and assessed using qPCR. Data are expressed as log2-normalized relative expression to the housekeeping gene β-actin. Snapshots of represented immunohistochemistry staining of the same kidney biopsies of DN and HC donors [as in panel **(A)**] using human-specific monoclonal antibodies against human IL-1α and IL-1β, panel **(B)** shows glomeruli, whereas panel **(C)** shows renal tubule. Upper panels show IL-1α, and lower panels show IL-1β. Brown staining indicates the presence of the target antigen. **(D)** Interleukin 1α expression is upregulated in response to HG concentrations. The human proximal tubular kidney cells HK-2 exposed to gradual glucose concentrations for 24 or 48 h. Interleukin 1α gene expression was evaluated using qPCR and expressed as normalized relative expression to the human housekeeping gene β-actin. **(E)** Interleukin 1β expression is not induced in response to HG concentrations. HK-2 cells were exposed to gradual glucose concentrations for 48 h. The expressions of IL-1α and IL-1β were monitored as in panel **(E)**. **(F)** HK-2 cells were exposed to low (5 mM), high (45 mM), d-glucose, or l-glucose (45 mM) concentrations for 48 h. The expression of IL-1α transcripts was evaluated using qPCR as in panel **(E)**. **(G)** The overexpression of IL-1α in response to HG concentration can be abolished by NF-κB inhibition. HK-2 cells were exposed to HG concentrations for 48 h with or without the presence of 5 μM/mL of BAY 11-7082 NF-κB irreversible inhibitor. The expression of IL-1α was monitored as in panel **(E)**. *t* test *p* values: *p < 0.05, **p < 0.01, ***p < 0.001.

To better understand the pattern and dynamics of the IL-1 genes in DN, we first tested the expression of a panel of IL-1 genes (e.g., IL-1α, IL-1β, and IL-1Ra) in biopsies taken from patients diagnosed with different stages of DKD. As control kidney tissue, we used biopsies taken from donors who had elective nephrectomy and did not have any record of diabetes mellitus or histological signs of inflammation. Total RNA was extracted from paraffin-embedded sections, and IL-1 gene expressions between the healthy control (HC) group and DN tissues were compared (Figure 1A). Surprisingly, in contrast to a recent report (20), we could not observe a substantial IL-1β expression in any of the biopsies normalized gene expression comparing DN to HC. Remarkably, a dramatic IL-1α expression could be seen, especially in DN samples, which shows significant tubulointerstitial injury followed by elevation in IL-1Ra (Figure 1A). To localize IL-1R agonistic proteins expression and in order to better understand their cellular source, we performed immunostaining on the same DN and HC biopsies. In total, we found that the protein expression assessed by immunostaining showed full correspondence with our IL-1 RNA gene expression (Figure 1A). Overall, both IL-1 cytokines were utterly absent from glomeruli, both in control and in the inflamed DN samples (Figure 1B). Although IL-1α showed strong cytoplasmic expression, especially in dilated atrophic renal proximal tubule cells of DN patients, no IL-1β could be detected in any of the kidney DN samples (Figure 1C). Remarkably, in agreement with our data, the recent report by Lei et al. (20) also shows extensive IL-1α immunostaining in biopsies of human DN of the tubular cells with marginal positive glomeruli staining present in the control samples. Importantly, IL-1α and not IL-1β transcript levels were shown to be elevated in a mouse model of diabetes mellitus type 2 significantly above wild-type (WT) mice, supporting the notion that IL-1β transcript and protein expression in the kidney predominately originate from infiltrating immune cells and not from any of the kidney cells (20).

Interleukin 1α is now widely accepted as an alarmin molecule released from damaged or necrotic cells as an initiator of sterile inflammation and tissue repair (17, 18, 21). Recently, this activity was further extended, showing that alarmins and, in particular, IL-1α can directly sense and report damage by signaling to the environment when released from live cells undergoing non-lethal physiological stress, a function denoted as stressorin (13). To further validate and establish our observation that IL-1α may play a role in promoting tubular inflammation in the DN, we tested the effect of HG, as non-lethal metabolic stress, on human proximal tubular cells using the HK-2 cells. Applying HG consecrations (30 and 45 mM vs. 5 mM normal glucose) leads to a gradual increase of IL-1α expression over time (Figure 1D). Following the expression results obtained from the renal biopsies, no induction of IL-1β transcript was detected in these conditions (Figure 1E). We additionally verified using the unnatural organic compound stereoisomer of d-glucose and l-glucose, which cannot be used as a source of energy, that the increase in IL-1α expression levels is a result of the elevated d-glucose concentrations (Figure 1F).

Because the perpetuated activation of NF-κB and elevated levels of several cytokines were demonstrated to play a critical role in damaging renal tubule (22), we tested the possibility that NF-κB activation may orchestrate the glucose-induced expression of IL-1α in renal tubular epithelial cells. Interestingly, NF-κB activation in tubular epithelial cells, together with the increased transcription of specific proinflammatory chemokines, was also suggested as markers of progressive DN (23). Indeed, inhibition of NF-κB using the inhibitor BAY-11-7082 abolished the increased expression of IL-1α in HG (Figure 1G).

**Figure 2:**
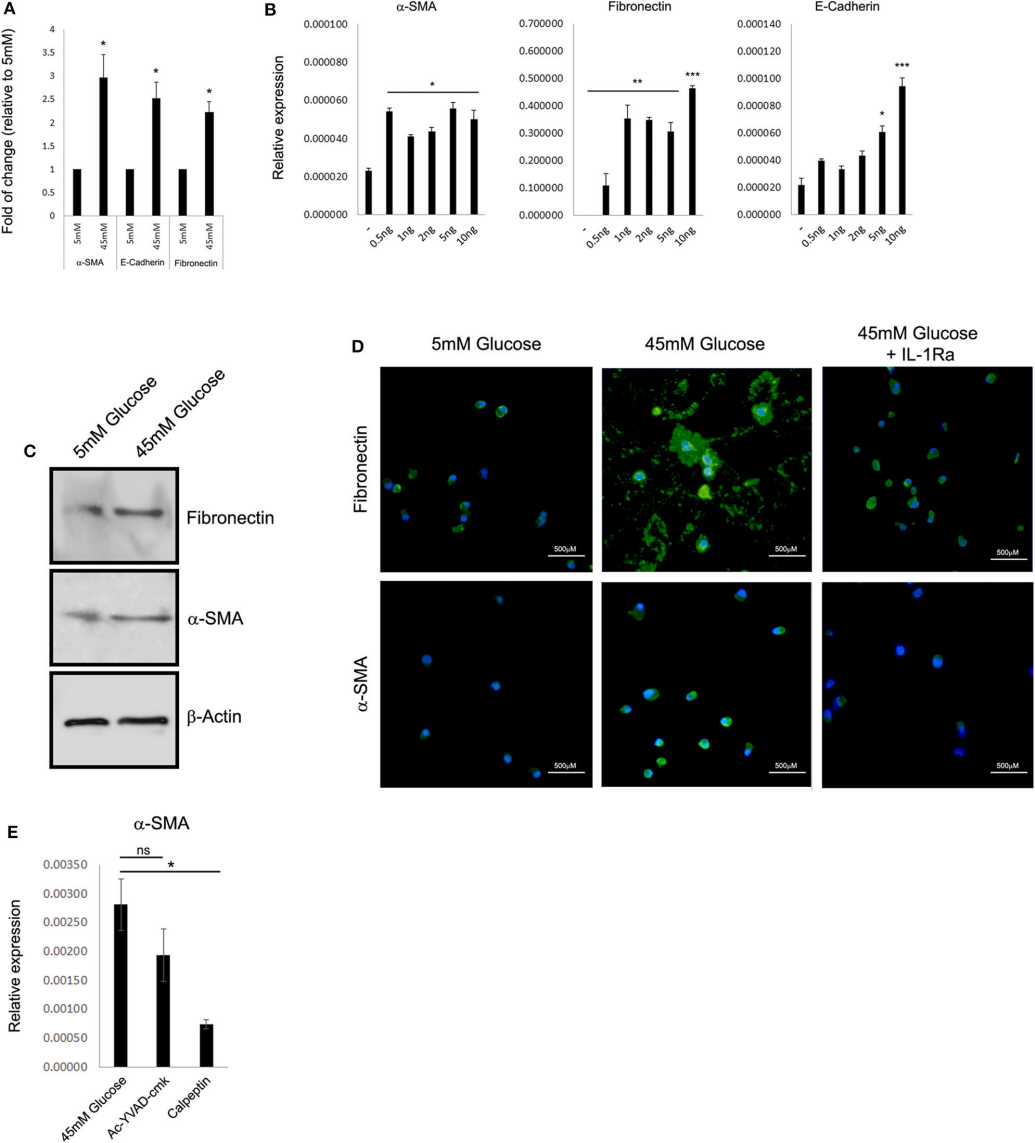
High glucose leads to IL-1α-dependent expression of extracellular matrix proteins in renal tubular epithelial cells. **(A)** HK-2 human proximal tubular kidney cells were exposed to different glucose concentrations for 48 h. The expression of the EMC genes α-SMA, E-cadherin, and fibronectin was evaluated using qPCR and was normalized relative to the expression of the housekeeping gene β-actin. Results are expressed as fold of change relative to the control of low glucose concentration (5 mM). **(B)** Gradual increasing concentrations of recombinant human IL-1α (rhIL-1α) were added to quiescent HK-2 cells in 5 mM low glucose. The expression of the EMC genes α-SMA, E-cadherin, and fibronectin was evaluated using qPCR as in panel **(A)** after 24 h. Data are expressed as normalized relative expression to β-actin. **(C)** Western blot of whole-cell proteins assessing the cell-associated levels of the EMC proteins α-SMA and fibronectin after exposer of HK-2 cells to low (5 mM), high glucose (45 mM). **(D)** Representative immunofluorescence snapshots of HK-2 cells in low (5 mM), HG (45 mM), or HG with rhIL-1α (10 ng/mL). Designated antibodies were used to detect the intracellular or extracellular localization and expression levels of the ECM proteins fibronectin and α-SMA. Green: fibronectin; yellow: α-SMA; and blue: DAPI; white lines show 500-μm bars. Note that the extracellular staining is the deposition of ECM proteins (fibronectin, middle panel). **(E)** HK-2 cells were exposed to HG for 48 h with or without the presence of Ac-YVAD-cmk (50 μM) or calpeptin (20 μM). The expression of α-SMA was monitored using qPCR as in panel **(A)**. *t* test *p* values: *p < 0.05, **p < 0.01, ***p < 0.001.

Previously, many reports indicated that renal function in DN correlates better with tubular changes than with glomerular pathology (22, 24, 25). One of the key features of advanced human DN that most rodent models lack is the development of significant interstitial fibrosis. Renal fibrosis is characterized by the pathological deposition of the uncontrolled build-up of the extracellular matrix (ECM). Renal ECM is a dynamic structure undergoing remodeling, particularly during fibrosis, and composed of a complex network of collagen, elastin, and several glycoproteins and proteoglycans forming basal membranes and interstitial space including vimentin, E-cadherin, α-smooth muscle actin (α-SMA), and fibronectin (26). From a clinical perspective, ECM proteins are directly involved in several renal diseases and indirectly in Chronic Kidney Disease (CKD) progression during renal fibrosis (26). Thus, we tested if HK-2 cells cultured under HG conditions can provide a model system for studying the effect of elevated glucose on the deposition of ECM. Forty-eight-hour exposure to HG levels resulted in elevated expression of the ECM proteins α-SMA, E-cadherin, and fibronectin (Figure 2A). Most importantly, exogenous addition of recombinant rhIL-1α in 5 mM low glucose leads to a comparable elevation of the following ECM genes, even in the absence of HG (Figure 2B). Interestingly, HK-2 cells seem to be highly sensitive to relatively low concentrations of rhIL-1α (0.5 ng/mL) signaling when especially the expression of fibronectin is dramatically induced by the ectopic addition of rhIL-1α. Next, we evaluated the levels of the EMC proteins α-SMA and fibronectin in whole-cell protein extracts of HK-2 exposed to low glucose or HG concentration. Western blot using specific monoclonal antibodies of α-SMA and fibronectin confirmed our previous observation and showed the elevation in the steady-state levels of both cell-associated ECM proteins (Figure 2C). Because HG upregulates fibronectin (27), and IL-1 treatment causes a fibrotic-like state in human HK-2 cells *in vitro* (28), we tested the possibility that the IL-1α-dependent primary intrinsic inflammation, which stimulates cells to adopt an activated state characterized by enhanced expression of ECM protein production, can be attenuated by IL-1Ra. In support of our hypothesis, when tested in an animal rat model, administration of an IL-1Ra almost completely abrogated tubular atrophy (29). We performed immunofluorescence of the ECM proteins α-SMA and fibronectin in human HK-2 cells exposed to 5 mM (low glucose), 45 mM (HG), or HG with the presence of 10 ng/mL recombinant IL-1Ra. While HG shows enhanced production and deposition of both fibronectin and α-SMA, the presence of IL-1Ra and blocking IL-1R signaling dramatically abrogated their overproduction and deposition (Figure 2D). Next, we tested if the cell-permeable Ac-YVAD-cmk selective and irreversible inhibitor of the cysteine protease caspase-1, also known as the IL-1β-converting enzyme or the cell-permeable calpain inhibitor calpeptin, affects the expression of ECM proteins induced by HG concentration. In correspondence with our previous results, the Ac-YVAD-cmk inhibitor had no significant effect on the expression of α-SMA after exposure to 48 h of HG, whereas the IL-1α calcium-activated proteases calpain inhibitor (calpeptin) leads to almost four times' significant reduction in the expression of α-SMA under the same HG conditions (Figure 2E).

Taken together, our data demonstrate that IL-1α and not IL-1β, is the major proinflammatory cytokine from the IL-1 family, which is expressed in the kidney in response to HG stress of DN patients.

## Discussion

In this work, we show that hyperglycemia induces tubular production of IL-1α that may contribute to the pathogenesis of tubulointerstitial injury in DN. Our findings are strongly supported by previous reports that IL-1α-889 C/T polymorphism (30) and IL-1 receptor antagonist allele (IL1RN^*^2) were associated with nephropathy in diabetes mellitus (31). Moreover, serum inflammatory cytokines and immune mediators were found to be elevated in early-stage DN such as IL-1α (and not IL-1β) in addition to g**ranulocyte**-macrophage colony-stimulating factor, IL-1Ra, IL-6, and MIP-1 and increase with CKD progression until stage 4–5, at which point a decrease was observed parallel to a loss of functional renal mass that occurs in late-stage CKD (32).

Emerging evidence supports a role for tubular involvement in DKD, whereas proximal tubular injury and dysfunction play a role in the early pathogenesis of diabetes affecting the kidney. Exposure of proximal tubular cells to HG concentrations and advanced glycosylation end products in diabetes increased the expression of different profibrotic cytokines and generation of intracellular reactive oxygen species (7). In patients with type 2 diabetes with nephropathy, tubular P2X4R (P2X4R purinoreceptor) expression is upregulated and closely related to NLRP3 inflammasome activation and renal interstitial inflammation (33). Proinflammatory cytokines can stimulate TGFβ1 synthesis by proximal tubular cells (34), which induce interstitial fibrosis and lead to histological tubulointerstitial changes that associate with the progression of DKD (35).

Our data show that the proinflammatory cytokine IL-1α is induced in the tubular cells but not in the glomerular area in patients with DKD with reduced GFR (Table 1). Likewise, exposure of human tubular cells to an HG concentration increased the expression of IL-1α in these cells over time, most probably a result of the metabolic stress and cellular injury. Thus, local production of IL-1α and autocrine IL-1 signaling during hyperglycemia and the following uncontrolled deposition of ECM proteins such as fibronectin and α-SMA may play a role in the progression of DKD by promoting or intensifying renal fibrosis.

Indeed, it was previously suggested that, inside the kidney, renal cells might initiate sterile inflammatory cascade by releasing alarmins during metabolic stress or damage. Therefore, further support for our findings can be found in the report that Nlrp3 inflammasome activation in non-myeloid-derived cells (most probably renal cells) aggravates DKD (16). Abolishing Nlrp3 or caspase-1 expression in bone marrow–derived cells fails to protect mice against DKD, and Nlrp3-deficient mice were protected against DKD despite transplantation of WT bone marrow (16) showing that inflammation originating from renal cells and not myeloid cells can be attributed to DKD progression and deterioration.

Despite the fact that little is known about the mechanisms underlying the activation and secretion of IL-1α, its release is clearly regulated also by the inflammasome (36). Accordingly, NLRP3 inflammasome agonists, such as uric acid crystal or nigericin, induce IL-1α cleavage and secretion, and depending on the type of NLRP3 agonist, the release of IL-1α is NLRP3 inflammasome–dependent followed by calpain processing (37). Interestingly, in support of our findings, inhibition of the IL-1α processing protease calpain protects mice from injury and renal inflammation, minimizing kidney vascular lesions related to aging (38). The fact that calpeptin mitigates α-SMA expression in response to HG suggests that IL-1α release from renal tubular cells is primarily active secretion as stressorin and not passively released by massive necrosis as an alarmin. Indeed, the inflammasome is long known as a “danger sensing” complex that triggers innate immunity when NLRs are a family of intracellular sensors of “danger signals” that have emerged as being crucial components of the innate immune responses and inflammation (39). Our results support a model in which IL-1α contributes to the pathogenesis of the kidney in DKD, probably as a result of chronic hyperglycemia (40) from renal tubular epithelial cells. In such cases, IL-1α release is more relevant inside the kidney and then IL-1β (41), whereas IL-1α is known to elicit sterile systemic inflammation and tissue repair (18, 21). Although we cannot rule out other factors such as high levels of albumin that were shown to act as DAMPs and stimulate tubular inflammation also via the activation of the inflammasome component Nlrp3 (42), we show that IL-1α expression is induced in these cells, probably as a danger or stress signal (13), as a result of HG. Moreover, it was previously shown that HG induces renal tubular epithelial injury via Sirt1/NF-κB signaling pathway (19), while the same cells show increased hyaluronan synthesis in response to either IL-1 or elevated d-glucose (25 mmol/L), which is associated with NF-κB–activated transcription of the HAS2 gene (43), and contribute to tubular injury and interstitial fibrosis in the kidney.

Therefore, blocking the inflammasome–IL-1α/IL-1R signaling cascade (41) may show a great therapeutic potential benefits in kidney injury, especially in DKD, and may help to attenuate disease progression to ESRD.

## Data Availability Statement

All datasets generated for this study are included in the article/Supplementary Material.

## Ethics Statement

This is a retrospective study that was conducted at the Galilee Medical Center, Naharyia, Israel after the approval of this institute's Helsinki Committee (protocol number 0067-17 NHR).

## Author Contributions

TS and IC performed RNA extractions, analysis of gene expression by qPCR, transcriptional analysis of human biopsies, and tissue culture-based experiments. RH performed immunostaining of human kidney biopsies. SC-P provides statistical support and analysis to all presented data and generated figures. IC, GB-S, and KK designed the study and were responsible for funding. IC, GB-S, and KK analyzed the data and wrote the manuscript. All authors revised and approved the final version of the manuscript.

## Conflict of Interest

The authors declare that the research was conducted in the absence of any commercial or financial relationships that could be construed as a potential conflict of interest.
