# Supplementary material for: Glucose Induces IL-1α-Dependent Inflammation and Extracellular Matrix Proteins Expression and Deposition in Renal Tubular Epithelial Cells in Diabetic Kidney Disease
Source: Front Immunol. 2020 Jul 7;11:1270. doi: 10.3389/fimmu.2020.01270 (PMC7358427; doi:10.3389/fimmu.2020.01270)
Supplement: Supplementary file 1 [file Data_Sheet_1.PDF]

Supplementary Table-1: Primers used in this study

Primer name	Sequence
Human IL-1α F	AAGATGAAGACCAACCAGTGC
Human IL-1α R	AACAAGTTTGGATGGGCAACT
Human IL-1β F	ATGATGGCTTATTACAGTGGCAA
Human IL-1β R	GTCGGAGATTCGTAGCTGGA
Human IL-1Ra F	GTCAATTTAGAAGAAAAGATAGATGTGG
Human IL-1Ra R	GTCCTGCTTTCTGTTCTCGC
E-Cadherin F	TACACTGCCCAGGAGCCAGA
E-Cadherin R	TGGCACCAGTGTCCGGATTA
Fibronectin F	CCATCGCAAACCGCTGCCAT
Fibronectin R	AACACTTCTCAGCTATGGGCTT
α-smooth muscle actin (a-SMA) F	ACTGAGCGTGGCTATTCCTCCGTT
α-smooth muscle actin (a-SMA) R	GCAGTGGCCATCTCATTTTCA
Human β-actin F	TGCCATCCTAAAAGCCACCCCACTTCTCTC
Human β-actin R	AAGCAATGCTATCACCTCCCCTGTGTGGAC

*Sequences are shown in 5'-3' when F-Forward and R-Reverse.
